# Phylourny: efficiently calculating elimination tournament win probabilities via phylogenetic methods

**DOI:** 10.1007/s11222-023-10246-y

**Published:** 2023-05-16

**Authors:** Ben Bettisworth, Alexander I. Jordan, Alexandros Stamatakis

**Affiliations:** 1 Computational Molecular Evolution, Heidelberg Institute for Theoretical Studies, Heidelberg, Germany; 2 Computational Statistics, Heidelberg Institute for Theoretical Studies, Heidelberg, Germany; 3 Institute for Theoretical Informatics, Karlsruhe Institute of Technology, Karlsruhe, Germany; 4 Institute of Computer Science, Foundation for Research and Technology - Hellas, Hellas, Greece

**Keywords:** Sports forecasting, MCMC search, Uncertainty analysis, Phylogenetic analysis

## Abstract

The prediction of knockout tournaments represents an area of large public interest and active academic as well as industrial research. Here, we show how one can leverage the computational analogies between calculating the phylogenetic likelihood score used in the area of molecular evolution to efficiently calculate, instead of approximate via simulations, the exact per-team tournament win probabilities, given a pairwise win probability matrix between all teams. We implement and make available our method as open-source code and show that it is two orders of magnitude faster than simulations and two or more orders of magnitude faster than calculating the exact per-team win probabilities naïvely, without taking into account the substantial computational savings induced by the tournament tree structure. Furthermore, we showcase novel prediction approaches that now become feasible due to this order of magnitude improvement in calculating tournament win probabilities. We demonstrate how to quantify prediction uncertainty by calculating 100,000 distinct tournament win probabilities for a tournament with 16 teams under slight variations of a reasonable pairwise win probability matrix within one minute on a standard laptop. We also conduct an analogous analysis for a tournament with 64 teams.

**Supplementary Information:**

The online version contains supplementary material available at 10.1007/s11222-023-10246-y.

## Introduction

Predicting the per-team win probabilities of a knockout tournament (alternatively bracket-based or elimination tournament) given a pairwise win probability matrix *P*, can become computationally expensive if a high degree of numerical accuracy shall be attained. In some cases the prediction will need to be computed thousands or even millions of times, for instance, to quantify the impact of slight perturbations of the pairwise win probability matrix *P* on the per-team tournament win probability. Given a tournament with *n* teams, one needs to evaluate a polynomial with $\approx 2^n$ terms to fully and exactly calculate the tournament win probability for a specific team via a naïve implementation (see the Sect. 2.1 for details). To calculate this tournament win probability for every team, an additional *n* such polynomials must be evaluated. Alternatively, one typically deploys stochastic simulations (again given a pairwise win probability matrix *P*), over the tournament tree to approximate the per-team win probabilities. Typically, this is computationally more efficient than computing the aforementioned polynomial, but comes at the cost of reduced numerical precision of the results (Ekstrøm et al. 2021; Demsyn-Jones 2019).

Prior work for predicting knock-out tournaments has generally focused on producing accurate outcomes, and not on the efficiency of the simulations per se. Consequently, these works generally deploy a statistical model of pairwise match win probabilities to predict match winners, such as the Bradley-Terry model, or an Independent Poisson Model which is also used in this work. Using such models, parameters are inferred from historic matches, and these parameters are subsequently used to predict the outcome of individual tournament matches (Ley et al. 2019; Groll et al. 2019). Alternatively, researchers have attempted to devise models for directly predicting the final ranking of teams in a tournament without taking into account the tournament (tree) structure (Tsokos et al. 2019). These models generally only infer a few sets of parameters, that is, only the most likely outcome is used to generate a prediction.

In the following, we propose a novel algorithm to efficiently ($O(n^2)$ which translates to a runtime improvement by 2–4 orders of magnitude) *and* exactly compute win probabilities for single elimination tournaments, given a square pairwise win probability matrix *P*. Our method was inspired by an observation by Yang (2006) that the Felsenstein Pruning Algorithm (Felsenstein 1981) can more generally be interpreted as an efficient way to compute polynomials of a high degree. We implement and make available our new method in an open source software tool named Phylourny (the name is a pun, on the words phylogeny and tournament). We experimentally demonstrate the order(s) of magnitude runtime improvement of Phylourny over stochastic tournament simulations and naïve evaluations of the polynomials. We also experimentally determine the differences in numerical accuracy between Phylourny and the stochastic simulation approach.

Finally, we showcase the new predictive possibilities that emerge through this increase in computational efficiency. By example of two recent tournaments, one with a large amount of data and one with a small amount of data (a basketball and football tournament respectively), we show how slight yet reasonable perturbations of *P* affect prediction uncertainty by calculating millions of tournament win probabilities within hours on a standard laptop. The main contribution of this paper is the substantially more computationally efficient approach to computing tournament win probabilities given a pairwise win probability matrix *P*. To this end, in our case studies we deploy a simplified version of a standard model from Ley et al. (2019) to compute *P* but do not propose improved approaches for computing *P*. Instead, we show to which extent slight alterations of *P* affect tournament win probabilities as such studies are now feasible in acceptable times with Phylourny.

## Methods

We initially describe our algorithm for exactly and efficiently calculating the tournament win probabilities in Sect. 2.1 and provide software implementation details in Sect. 2.2. Thereafter, we describe our simple models for calculating reasonable *P* matrices in Sect. 2.3 and outline how we deploy Markov Chain Monte Carlo (MCMC) sampling in Sect. 2.4 to quantify the prediction uncertainty induced by slight alterations of *P*.

### The Phylourny algorithm

We initially provide some definitions and introduce some notation.

**Fig. 1:**
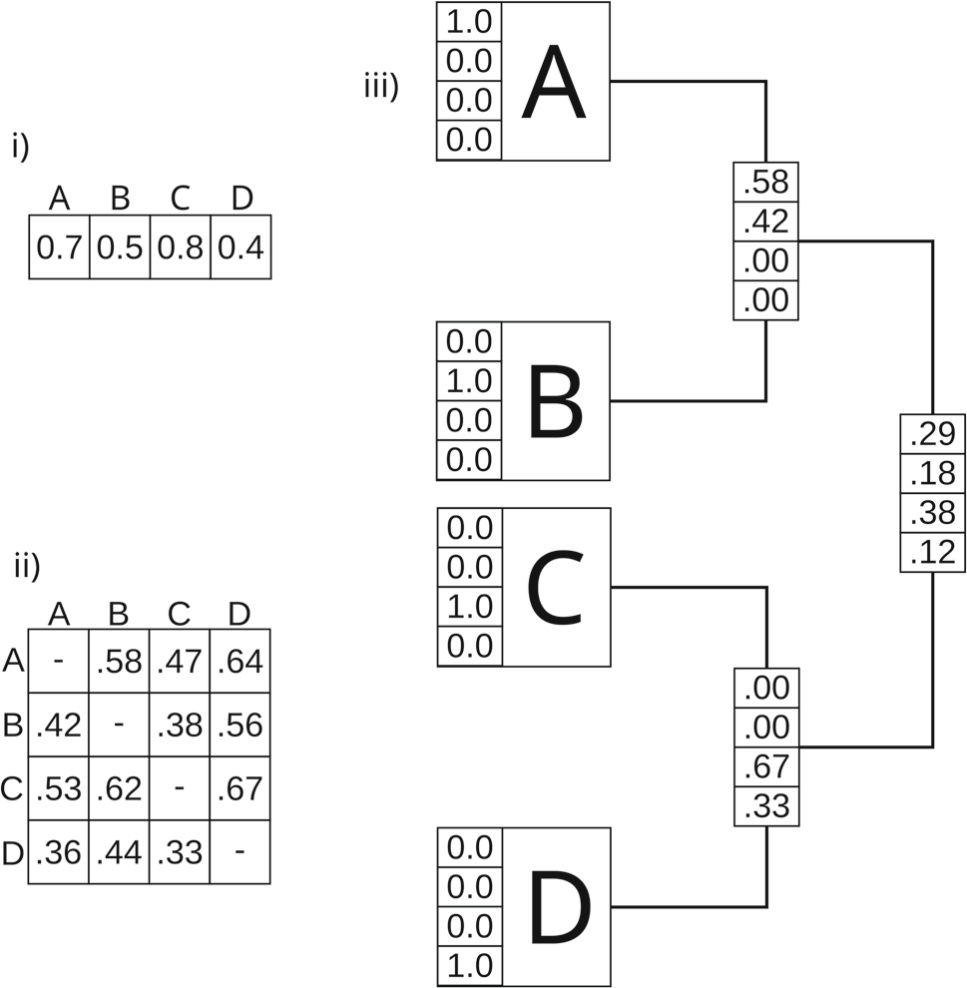
A worked example of a single elimination tournament with $n:=4$ teams. (i) A set of example team strength parameters. (ii) The *P* matrix created from the team strength parameters $r_a$ and $r_b$ using a simplified likelihood model, where the probability of “team *a* beats team *b*” equals $r_a/(r_a+r_b)$. (iii) A tournament with computed Win Probability Vectors (WPVs)

The win probability vector (WPV) for a given node in the tournament tree is a vector containing the probabilities of observing a specific team at that node, denoted by $R \in [0, 1]^n$, where *n* is the number of teams. Evidently, all tournament tree nodes below the tournament final, that is, the root of the tree, will comprise some entries that are equal to zero with the leaves being represented by the canonical unit vectors. For an illustration, see panel (iii) in Fig. 1.

Let $P_{a \vdash b} \in [0, 1]$ denote the pairwise probability of team *a* winning over team *b* in a single match, that is, the probability that “team *a* beats team *b*". By convention, we define $P_{a \vdash a} = 0$ for any team *a*. In the simplest tournament with only two teams, *a* and *b*, there is only a single match. The pairwise win probability matrix is given by

$$\begin{aligned} P = \begin{pmatrix} P_{a \vdash a} &{} P_{a \vdash b} \\ P_{b \vdash a} &{} P_{b \vdash b} \end{pmatrix}, \end{aligned}$$

and the WPV for the single node in this tournament is

1 $$\begin{aligned} R = (R_a, R_b) = (P_{a \vdash b}, P_{b \vdash a}). \end{aligned}$$

Because this constitutes a trivial case, the calculation is straight-forward. To recursively extend this to larger trees, we rewrite the above expression by also using the respective child nodes. First, we introduce the child WPVs as $V = (1, 0)$ and $W = (0, 1)$ leading to the expression

2 $$\begin{aligned} R_a = (P_{a \vdash a} \times W_a + P_{a \vdash b} \times W_b) \times V_a \end{aligned}$$

for the win probability $R_a$ of team *a*, assuming the team can only enter the match via the first child of the node as indicated by $V_a = 1$ and $W_a = 0$. As such, $P_{a \vdash a}\times W_a$ vanishes regardless of the value of $P_{a \vdash a}$, and Eq 2 reduces as given in Eq 1.

In general, for any number of teams *n*, the WPV *R* at any given node can be calculated from the respective child node WPVs *V* and *W* and the pairwise win probability matrix *P* as

3 $$\begin{aligned} R = V \odot \left( WP^\top \right) + W \odot \left( VP^\top \right) , \end{aligned}$$

where $\odot$ denotes the element-wise product. Please note that Eq. 3 is a generalized restatement of Eq. 2 using matrix and vector notation, and accounts for any team entering the match via either child. For single elimination tournaments at most one of $V_a$ and $W_a$ can be positive. The WPV at the root can be efficiently computed via a post-order traversal of the tournament tree, that is, by computing WPVs at the nodes bottom-up from the tips/leaves toward the final/root. Figure 1 depicts a simple example with the pairwise win probabilities represented as normalized relative team strengths.

In some tournaments, $P_{a \vdash b}$ will correspond to a “best of *k*" series of play-off matches instead of a single match, as for example in the National Basketball Association (NBA) playoffs. Further, this *k* can vary over the duration of the tournament since early matches are often “best of 1" with $k:=1$, whereas later matches might be “best of 5" with $k:=5$. We can seamlessly account for this by introducing *P*(*k*), a node-dependent pairwise win probability matrix for a “best of *k*” series.

### The Phylourny software

The open-source SPSVERBc1 implementation of our algorithm is available via GitHub at https://github.com/computations/phylourny under GNU GPL version 3.0. The software only requires SPSVERBc2 to build and git to download. Phylourny also implements stochastic (that is, simulation based) as well as naïve polynomial tournament win probability calculations for the sake of conducting run time and numerical precision comparisons. Finally, it offers the simple models for devising reasonable *P* matrices and conducting Markov Chain Monte Carlo (MCMC) sampling presented in the following Sects. 2.3 and 2.4. Finally, Phylourny has a software quality score of 7.7 as rated by the software quality analysis tool SoftWipe (Zapletal et al. 2021), which places Phylourny in the top 10% of scientific software tools included in the SoftWipe benchmark. Version v1.2.1 was used to perform the uncertainty analyses presented here.

Computing the *P* matrix based on the Poisson likelihood model (which is discussed in the next section) is comparatively computationally expensive. Therefore, to expedite these computations, we parallelized the computation of the likelihood over the historic matches using OpenMP (Open 2020). Despite this parallelization, the computation of the likelihood score still accounts for approximately 90% of the overall run time of the Poisson model based MCMC analysis.

To perform analysis with Phylourny, a list of teams who will participate in the elimination tournament needs to be provided as a file. Phylourny then can compute a win probability when given probability matrix, which should be provided as a CSV file. Alternatively, Phylourny can conduct an MCMC search of the parameter space of the Independent Poisson Likelihood Model (discussed in Sect. 2.3). In this case, a list of historical matches needs to be provided in a CSV file. The results from the MCMC search will be summarized in 3 output files with three different summaries: the maximum likelihood prediction (MLP) which is the prediction using the parameters with the highest likelihood; the maximum marginal posterior prediction (MMPP) which is the prediction averaged over all posterior samples; and the list of samples taken from the posterior during the MCMC search. The MLP and MMPP are discussed in more detail in Sect. 2.4.

### The independent Poisson likelihood model

The success of a tournament prediction heavily relies on the *P* matrix, that is, the methods used to calculate and also the data used to evaluate its likelihood. Thus, improved methods for obtaining this matrix constitute an active area of research but improving upon them is beyond the scope of this paper (Kaplan et al. 2014; Hill 2021; Hvattum and Arntzen 2010; Lock and Nettleton 2014). As a simple yet effective reference model, we adapt the “Independent Poisson Model” from Ley et al. (2019) to model the pairwise win probabilities based on historical match data. In a nutshell, two competing teams are assumed to independently score points under respective Poisson distributions, with parameters driven mainly by the difference of the teams’ strengths. The win probability of a team is the probability to score more points than the opponent as given by the Skellam distribution that describes the difference between two independent Poisson random variables.

Our version of the Independent Poisson Model is a straightforward implementation of the model described in Ley et al. (2019), slightly modified by removing the constraint that the team strength parameters need to sum to zero. During our MCMC search, we constrain the strength parameters to be between 0.0 and 1.0, which has a similar effect. Additionally, we remove the distinction between home and away games to further simplify the model. A home advantage parameter could be integrated into the model in a future version of Phylourny. Let *M* denote a series of historical matches $(a,b,g_a,g_b)$, where *a* and *b* are the teams and $g_a$ and $g_b$ are the goals scored by each team, respectively. Then, the likelihood of the Independent Poisson Model is given by

4 $$\begin{aligned} L(R, \rho ) = \prod _{(a,b,g_a, g_b) \in M} \left( \frac{\lambda _{a\vdash b}^{g_a}}{g_a!} e^{-\lambda _{a \vdash b}} \times \frac{\lambda _{b \vdash a}^{g_b}}{g_b!} e^{-\lambda _{b \vdash a}} \right) , \end{aligned}$$

where $R = (r_a, r_b, \dots ) \in [0, 1]^n$ is the parameter vector of team strengths that reflect the skill levels of each team, and $\rho \in \mathbb {R}$ represents an “average” skill level among all teams in the Poisson parameter

$$\begin{aligned} \lambda _{a \vdash b} = e^{r_a - r_b + \rho }. \end{aligned}$$

The expression in Eq. 4 is useful to describe the model. However, it is unsuitable for computation in general as many sports have score counts which are substantially larger than that of football. For example, basketball scores are generally 10–80 times higher. The issue is that when scores are large, some terms in the computation simultaneously become very large (for example $g_a!$) and very small (for example $e^{-\lambda _{a \vdash b}}$). This introduces substantial numerical deviations, which can potentially be amplified by the MCMC search, as it might sample numerical error under unfavorable conditions. If numerical deviations yield likelihood scores that are better than the exact analytical likelihood scores, the MCMC search will preferably sample points in parameter space that maximize the numerical error. While a strong prior can prevent this in many cases, it is preferable to devise more numerically stable computations, to prevent this type of potential error a priori. To alleviate this, we deploy the standard solution to reduce numerical error by computing the log-likelihood instead. As we show, this provides sufficient numerical stability to also apply this model to basketball.

Additionally, the particular model we use for the sake of the example, might likely not be correct for many sports, including basketball. This is because a Poisson distribution always has a mean equal to its variance. However, this assumption does likely not hold for sports such as basketball, where the score variance is generally much smaller than the score mean. For example, in the dataset for the basketball tournament we analyze later in this work, the mean score is $\approx 70$ and the standard deviation is $\approx 12$. Nonetheless, we choose to use the Independent Poisson Model as it strikes a good balance between realism and simplicity to substantiate our claims that novel types of statistical analyses are feasible because of the computational savings of Phylourny.

While we do present and implement as open-source code the Independent Poisson Model here and use this model for MCMC analyses (see below), Phylourny does by no means rely on this particular model. In fact, any model which can compute a pairwise win probability matrix *P* can be used. Furthermore, any parametric model can be used to perform the MCMC analyses we describe next.

### Sampling the P matrix via MCMC

**Fig. 2:**
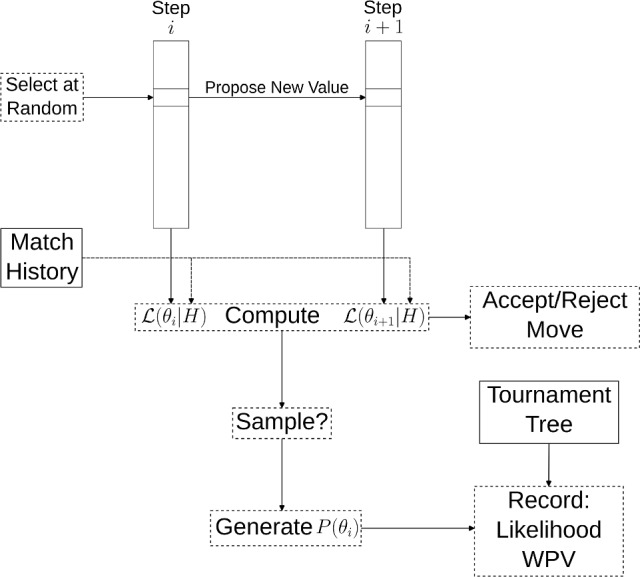
Diagram showing an MCMC step for Phylourny. The function $\mathcal {L}(\cdot \vert H)$ denotes the likelihood function and $\theta _i$ denotes the parameters of the model for the matrix *P*. For the Independent Poisson model, $\theta$ comprises *R* and $\rho$ and $\mathcal {L}(\theta \vert H) = L(R, \rho )$ from Eq 4. The decision whether to accept $\theta _{i + 1}$ depends on the prior and the proposal distribution in addition to the likelihood. Solid borders represent inputs to the algorithm, while dashed borders steps of the algorithm

To generate a sample of reasonable *P* matrices that accurately reflect a given match history and to quantify the uncertainty of the tournament win probabilities at the WPV of the final, we deploy MCMC sampling via the Metropolis-Hastings algorithm (Metropolis et al. 1953). A diagram showing an example sampling step is given in Fig. 2.

At each MCMC step, a new set of model parameters for the Independent Poisson model is proposed yielding a new pairwise win probability matrix $P'$, and the likelihood of these parameters is computed, i.e. $L(R, \rho )$. If the proposed model parameters are accepted, then the WPV of the tournament is computed under $P'$ and recorded as a sample together with the likelihood of the corresponding model. Optionally, the sample set can be thinned for saving disk space by taking a sample only every *n* generations.

At the start of the MCMC chain, all parameters are initialized to 0.5. In each MCMC step, a parameter is selected at random with equal probability. If a team strength *r* is selected, then a new strength $r'$ is proposed according to a Beta distribution with $\alpha := \beta := 1.5$. The corresponding density function is denoted by $b_{1.5}$. If the average strength $\rho$ is selected, then a new average strength $\rho$ is proposed by adding a value drawn from a Normal distribution with $\mu := 0.0$ and $\sigma := 0.1$, or simply $\rho ' \sim \mathcal {N}(\rho , 0.1^2)$. Both proposal functions have no particular meaning, and could be replaced with other proposals so long as they satisfied a few requirements. First, the average skill level $\rho$ must be allowed to vary to any value in $\mathbb {R}$. Second, the strength parameters can be shifted, as a group, by some constant and still have the same likelihood. Therefore, to improve convergence of the chain, we found it best to constrain the strength parameters by using a proposal with a single mode, which has the effect of preferring an average relative strength of 0.5. We could have implemented this as a prior with the same requirements on the strength parameters, however we found it simpler to satisfy these requirements with the proposal than to implement these requirements as a prior distribution.

The proposal process is symmetric in the average strength, but non-symmetric in any team strength, so we calculate the Hasting’s ratio as 1 and $b_{1.5}(r)/b_{1.5}(r')$, respectively. The acceptance ratio is then computed as the product of the likelihood ratio, the prior ratio, and the Hasting’s ratio. A value for the acceptance ratio larger than 1 is reduced to 1. We accept a proposed new team strength with a probability that is equal to the acceptance ratio. We implemented and tested several priors including a Normal distribution, a Beta distribution, and an Uniform Distribution. None of the priors had a strong effect on the results, so we chose to use a Uniform prior, for the sake of simplicity. Finally, we sample the chain every 100 generations in order to thin the samples. Thinning is performed so that some result files, particularly the file containing the samples, do not become excessively large.

The MCMC sampling procedure should be continued until the chain has reached “apparent convergence" as true convergence can only be attained if the MCMC sampling is executed infinitely. Further, only the lack of convergence can be assessed via appropriate diagnosis tools. Hence, as assessing the convergence of MCMC is impossible, in our experiments, we only draw a fixed number of samples. However, computing the WPV of a single sample using Phylourny is computationally inexpensive. Therefore, we are able to compute an extremely large number of samples within an acceptable amount of time. For a football tournament with $n:=16$ teams (the UEFA 2020 knock-out stage), we can evaluate 10 million proposals under the Independent Poisson model which result in exactly 100 thousand WPV samples after thinning, within approximately 51 seconds using a standard laptop. This corresponds to approximately 1961 exact calculations of the tournament final WPV and 196, 078 likelihood evaluations per second. We believe that using 100 thousand samples is justified, as the state space for *this* specific tournament is not excessively large, and should be sufficiently sampled with this number of samples, particularly since we explore the parameter space for $\approx 10$ million generations.

We discard the first 10 thousand samples (10% of samples) as burn in to compute summary statistics. Once we have obtained all sampled WPVs from the MCMC procedure, we can compute two predictions: the maximum likelihood prediction (MLP), or the maximum marginal posterior prediction (MMPP). The MLP is simply the prediction given by the *P* matrix that yielded the highest likelihood score, whereas the MMPP is the average prediction over all samples. Because an MCMC procedure will sample the posterior with a probability distribution hopefully approximating the true posterior, the average over all samples is approximately the average of the posterior. The difference between these two predictions is one of philosophy rather than mathematics, as they encapsulate distinct interpretations about what “really" matters. The school of thought advocating the MLP, claims that the only thing that matters is the *most likely* outcome, regardless of the underlying distribution, whereas the school of thought supporting the MMPP claims that the *totality of evidence* is what matters.

## Case studies, experimental setup, and hardware

We showcase and assess the runtime and the numerical performance of our method on two historical tournaments. We apply Phylourny to the 2020 UEFA European Football Championship (UEFA 2020) and the 2022 NCAA Division I Men’s Basketball Tournament (NCAA 2022) to perform an uncertainty analysis on the tournament results. As input to Phylourny we use historical match data from games played prior to the elimination phase to conduct MCMC searches, as described in Sect. 2.4.

We chose the UEFA 2020 and NCAA 2022 tournaments for several reasons which we think best allows us to showcase our method. First, UEFA 2020 and NCAA 2022 cover different sports, which allows us to show that Phylourny is not dependent on a specific sport. Second, UEFA 2020 and and NCAA 2022 have very different sizes, as UEFA tournaments have a small number of competitors (typically 16 teams) while NCAA tournaments have a large number (64 teams). Finally, the amount of historic match data available for NCAA tournaments is generally much more extensive than that of UEFA tournaments. Therefore, UEFA 2020 is the “small" case and NCAA 2020 is the “large" case. These two cases represent the extremes of tournament configuration in terms of size and matches before the tournament, and therefore they allow us to explore the entire range of Phylourny’s performance.

All input data and relevant output files of Phylourny for the experiments that we describe in more detail below are available at https://github.com/computations/phylourny.

### UEFA 2020 and NCAA 2022 historical match input data

We used the group stage matches for UEFA 2020 to perform our analysis. These matches are played in order to determine the “seeding" for the knockout round. In order to support UEFA 2020 representing the “small" case, we elected to *not* include qualifying round data, which are the matches played in order to determine who will enter the group stages. There were a total of 37 games, including tie breaker matches, which we included as historical match data in our analysis.

Because there is a more extensive pre-season to what is colloquially referred to as “March Madness" in the U.S. when compared to qualifying rounds for football tournaments, there is a more extensive dataset we can use for likelihood calculations. Therefore, a total of 1795 matches were eligible, that is involving at least one team which participated in the NCAA 2022 tournament, for use in our uncertainty analysis.

### MCMC analyses

**Fig. 3:**
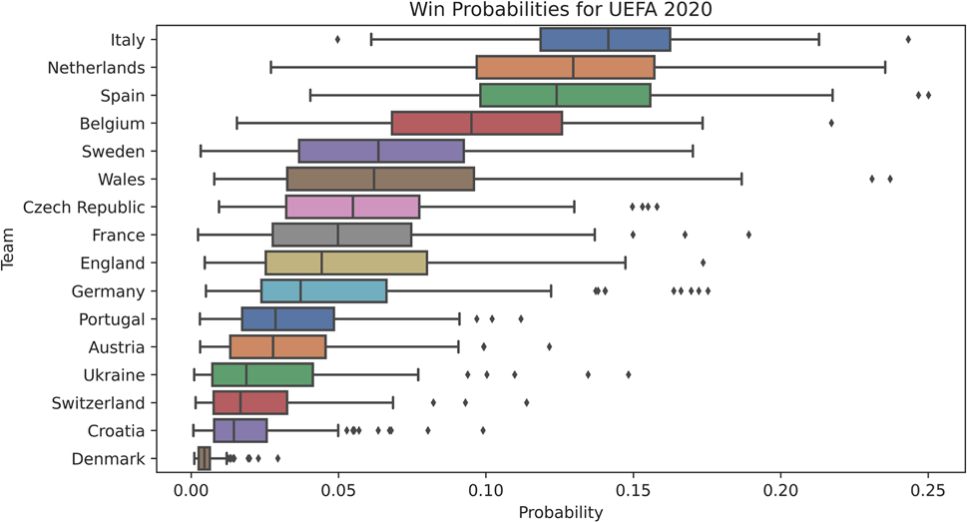
Probabilities for each team winning the UEFA 2020 tournament. The samples summarized are the top 0.1% percent of samples by likelihood from our 100,000 MCMC samples

**Fig. 4:**
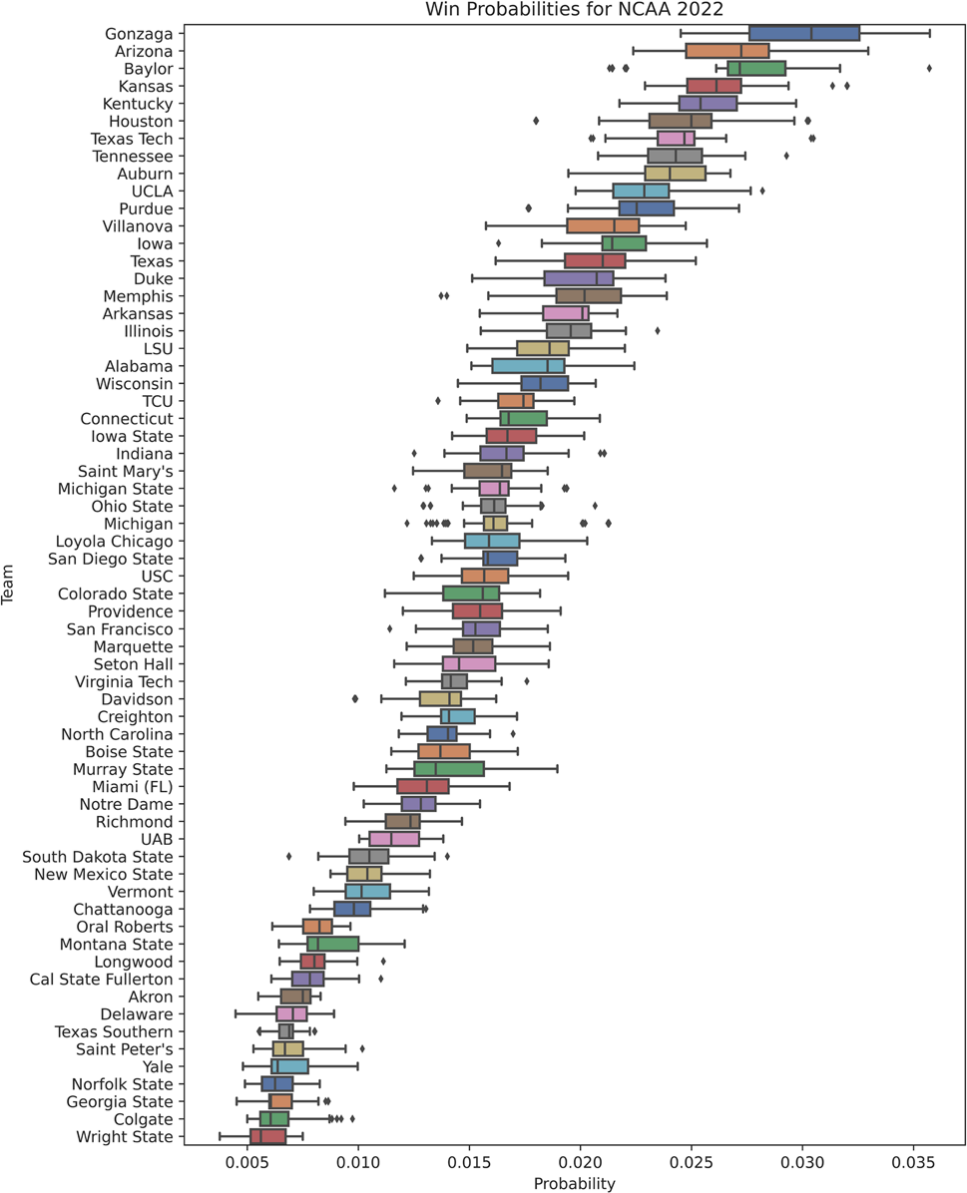
Probabilities for teams winning the NCAA 2022 tournament. The samples summarized are the top 0.1% percent of samples by likelihood from our 100,000 MCMC samples

As described in Sect. 2.4, the search was conducted via the Metropolis-Hastings algorithm (Metropolis et al. 1953). For the UEFA 2020 and NCAA 2022 uncertainty analyses, 100,000 samples were collected with thinning enabled. We present summary statistics for the most likely of these samples (the 99.9%-ile) for the UEFA 2020 and NCAA 2022 analyses in Figs. 3 and 4, respectively.

### Hardware used and build parameters

We used a Intel i7 CPU with 4 cores clocked at 2.8 GHz with 16 GiB of memory for all computational experiments. We used GCC version 12.1.1(GCC 2022) and CMake version 3.23.3 to build Phylourny. Phylourny was built using CMake’s “Release” mode, which removes most debug information. Phylourny itself was built as version v1.2.1 and was built and executed for the purposes of analysis on Linux 5.18.16.

### Numerical error assessment

We also investigate the numerical error when using simulations to compute a WPV. To this end, we produced a sample of 1000 *P* matrices from an MCMC search for each of the two tournaments. The *P* matrices were sampled uniformly from the respective MCMC chains, after discarding the first 10% of samples as burn-in. For each sampled *P* matrix, we compute both the exact WPV using Phylourny, as well as an estimate using one hundred, one thousand, ten thousand, one hundred thousand, and one million simulations. For these estimates, we report both the relative error, which is

$$\begin{aligned} \text {Mean} \left( \left\| \frac{ \text {WPV}_{ \text {sim}, i } - \text {WPV}_{\text {phy}, i} }{ \text {WPV}_{\text {phy},i} } \right\| \right) \end{aligned}$$

where $\text {WPV}_{\text {sim}}$ is the WPV computed using simulations and $\text {WPV}_{\text {phy}}$ is the WPV computed using Phylourny. We also report the norm error, which is

$$\begin{aligned} \frac{\Vert \text {WPV}_{\text {sim}} - \text {WPV}_{\text {phy}} \Vert }{\Vert \text {WPV}_{\text {phy}}\Vert }. \end{aligned}$$

**Fig. 5:**
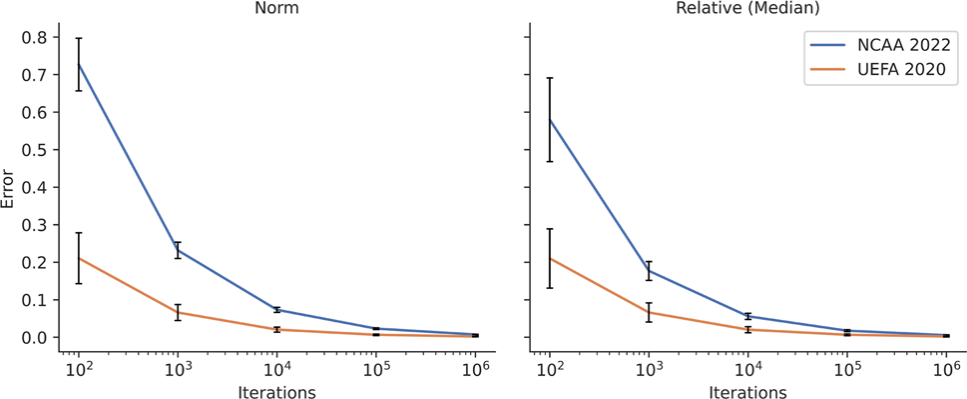
Plot of simulation errors with respect to number of simulations conducted for NCAA 2022 and UEFA 2020. We sampled 1000 *P* matrices from an MCMC search for each tournament. Error bars represent 1 standard deviation

The results from these analyses are summarized in Table 1 and in Fig. 5.

Additionally, we also conduct the same uncertainty analysis as described in Sect. 2.4 for both the UEFA 2020 and NCAA 2022 tournaments, but using simulations to estimate the WPVs instead of Phylourny. Results from these analyses are presented in two plots in the supplementary material.

**Table 1:** Simulation error for computing a WPV with an increasing number of samples for NCAA 2022 and UEFA 2020

Dataset	Simulation samples	Median relative error	Norm error
	100	0.727	0.691
	1000	0.232	0.216
NCAA 2022	10,000	0.073	0.069
	100,000	0.023	0.022
	1,000,000	0.007	0.007
	100	0.211	0.273
	1000	0.066	0.086
UEFA 2020	10,000	0.020	0.027
	100,000	0.007	0.009
	1,000,000	0.002	0.003

We used a sample of 1000 *P* matrices from an MCMC search for each tournament. Matrices were randomly sampled at uniform from an MCMC chain after discarding the first 10% as burn-in. In this table we report the mean of the 1000 samples

### Run time comparison

**Fig. 6:**
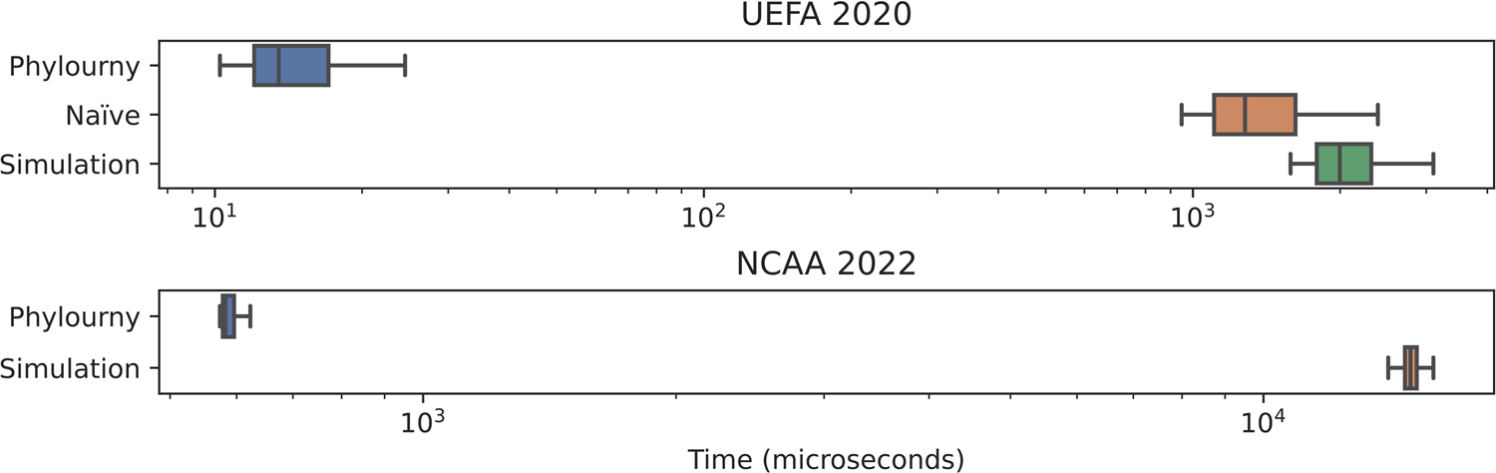
Tournament evaluation times for the different computation methods for 1000 sampled *P* matrices. Matrices were sampled according to the procedure described in Sect. 3.4. Times are reported as $\mu$s with a log scale. UEFA 2022 Simulation mean: 2111 $\mu$s, Naïve mean: 1413 $\mu$s, Phylourny mean: 15 $\mu$s. NCAA 2022 Simulation mean: 15010 $\mu$s, Phylourny mean: 599 $\mu$s. We did not obtain a time for the Naïve mode using NCAA 2022 as the time required to compute even a single evaluation was prohibitive. 1000 simulations were conducted for these run time measurements

Finally, we also compare the runtimes of Phylourny with other methods (simulations and naïve computation) for computing the tournament WPV. Using the sample of 1000 *P* matrices produced in Sect. 3.4 we also recorded the execution time for each method: Phylourny, Naïve, and Simulations. For comparison we only use 1000 simulations, which corresponds to a norm error of $>0.1$ on the UEFA 2020 dataset (see Table 1). While 1000 simulations are fewer simulations than one would utilize in a rigorous analysis, it is an appropriate choice as even this inaccurate level of simulation is less time efficient than Phylourny. The results from these runtime experiments are summarized in Fig. 6.

## Results

The analysis of the UEFA 2020 tournament with 16 teams required 51 s for 100,000 samples (generated via 10,000,000 MCMC steps), by executing the parallelized Poisson likelihood model using 4 cores on our test hardware system. The likelihood model calculations accounted for 90% of overall runtime. The analysis of the NCAA 2022 tournament required $\approx 1.5$ h for 100,000 samples (generated via 10,000,000 MCMC steps) and also using 4 cores. The difference in runtime is due to the substantially larger amount of data ($\approx 48$ times more historical match data when compared to the UEFA 2020 analysis) used to compute likelihoods for NCAA 2022. Approximately 80% of the runtime increase can be attributed to the larger historical match dataset used. In addition, there are 64 instead of 16 teams in the NCAA tournament, which increases the time required to compute tournament WPVs and the *P* matrix. The NCAA 2022 MCMC search achieved an acceptance ratio of $\approx .17$, while the UEFA 2020 MCMC search achieved an acceptance ratio of $\approx .67$.

**Table 2:** Summary statistics for the samples from the uncertainty analysis

	Mean	STD	Min	Median	Max
UEFA	$-$98.92	0.27	$-$99.22	$-$98.99	$-$97.91
NCAA	$-$13800	1.99	$-$13802	$-$13801	$-$13794

Values shown are Log-Likelihoods of samples taken during the MCMC search for the UEFA 2020 and NCAA 2022 uncertainty analysis which have been restricted to the 99.9%-ile

In Figs. 3 and 4 we summarize the results of the uncertainty analyses using the thinned samples from the MCMC search. We plot the results from their respective analyses, restricted to the top 0.1% (i.e., top 100 WPVs by log- likelihood) of samples by likelihood for UEFA 2020 and NCAA 2022. Additional summary statistics for these top 0.1% samples are shown in Table 2. Summary statistics for the entire thinned sample from the MCMC search set are presented in the supplementary materials.

Notable results include the correct identification of the winner for UEFA 2020 (Italy) and the high ranking for NCAA 2022 winner (Kansas) tournaments, who both receive a high median win probability in the uncertainty analysis. This is shown in Fig. 3 for UEFA 2020 and in Fig. 4 for NCAA 2022 probabilities.

We present the run times of three methods of computing WPVs in Fig. 6. Our method, Phylourny, performs the best (mean runtime 15 $\mu$s and 599 $\mu$s for UEFA 2020 and NCAA 2022 respectively). For smaller tournaments like UEFA 2020 we also found that it was faster to evaluate win probabilities naïvely (1413 $\mu$s) rather than conduct 1000 simulations (2111 $\mu$s). However, this does not hold for larger tournaments like NCAA 2022, where we were unable to obtain a result for the naïve computation, even after 2 h ($\approx 7.2 \times 10^9 \mu$s) of run time, whereas conducting 1000 simulations was feasible (15010 $\mu$s). To obtain these runtimes, we conducted 1000 simulations, which corresponds to a median relative error of $\approx 7\%$ in the case of small tournaments like UEFA 2020. Of all the methods tested here, Phylourny remains the least computationally expensive by $\approx 2$ orders of magnitude.

## Discussion

We have shown that the problem of predicting tournament winners is sufficiently similar to phylogenetic likelihood calculations such that analogous computational techniques can be applied. We have demonstrated this by developing methods inspired by computational phylogenetics to predict tournaments, and that applying these methods yields substantial computational speedups. In addition, we can calculate the final WPV of a tournament *exactly*, instead of using simulations to approximate it. This also allows, for instance, for a seamless deployment of MCMC methods as illustrated by our uncertainty analysis examples for the UEFA 2020 and NCAA 2022 tournaments.

Finding the appropriate method to infer an accurate pairwise win probability matrix *P* remains a challenge. Modeling sports in a way that will accurately determine the probability of a specific outcome is difficult. Private industry (bookmakers) as well as academic researchers have invested considerable effort into methods to predict the outcome of sports matches (Kaunitz et al. 2017; Lopez et al. 2018). These investigations are beyond the scope of our work, and we intentionally do not address more complicated pairwise win models. Instead, we have showcased that comparatively simple models, such as the Independent Poisson Model which was further simplified from its form in Ley et al. (2019), perform well when the uncertainty of the estimated model parameters is taken into account.

One may also argue that using the same *P* matrix through all stages of the tournament constitutes a simplification. In reality, the probability of a team beating another team most likely does not remain constant in the course of a tournament. Additionally, win probabilities might not remain constant for all matches in a “best of *k*” series. For some sports, particularly in the rising field of e-sports, adapted strategies will develop over the course of a series of repeated matches between two teams.

Despite these two (over-)simplifications, the ability to compute a WPV for a tournament both exactly and efficiently is highly useful, as advanced methods of analysis normally will require an exact result in order to be applicable. For example, when sampling from a posterior using an MCMC search using a complex model, it is desirable to have an accurate result for each sample, as this reduces the number of samples required to produce an accurate estimate of the posterior. While a sufficient degree of accuracy can be obtained via an appropriately large number of simulations, this approach is computationally expensive and will eventually become prohibitive. (Fig. 5)

Case in point, using the execution times measured in Fig. 6, the uncertainty analysis for NCAA 2022 which took approximately 1.5 h with Phylourny would have taken approximately 5 h using 1000 simulations per sample. However, in this case, 1000 simulations would correspond to a median relative error of $\approx 22\%$. To achieve a more acceptable error level, 10,000 simulations could be used per sample, but this would increase the expected runtime to $\approx 40$ h. We demonstrate that we can efficiently conduct such an analysis by implementing our own comparatively simple MCMC analysis of the UEFA EURO 2020 football tournament.

Additionally, Phylourny is model agnostic, which allows for more complicated models to be implemented. An example is to add a time element to the Independent Poisson Model, which increases the likelihood contribution of more recent matches when compared to older matches. In fact, this time element might be an accidental reason why our prediction of the UEFA 2020 is accurate, as we only include the group stage matches where Italy performed surprisingly well, as opposed to the extended match history including the qualifying round matches. Of course, this was not intentional but was an incidental result of limiting the historical data. Nonetheless, it shows how a likelihood model which incorporates match time would be advantageous. The likelihood model can also be augmented with the inclusion of match locations, which models a home game advantage and incorporates this advantage into the likelihood. A match location augmented likelihood model would also have implications for WPV computation, as the inclusion of location information for each match in the knockout round might improve results.

The main contribution of our work consists in the introduction of the *computational* method, which accelerates the exact computation of final win probabilities, given an estimate *P* of pairwise win probabilities, and the surprising connection between two seemingly unrelated branches of science.

We further demonstrate the efficiency and utility of Phylourny by implementing our own uncertainty analysis for the UEFA 2020 tournament as well as for the NCAA 2022 tournament. As can be seen in Figs. 3 and 4, there is a remarkable diversity of predicted outcomes. This is *despite* the likelihoods for these samples being essentially equivalent, as can be seen in Table 2. For example the difference between the minimum log-likelihood and the maximum log-likelihood for the 99.9%-ile samples for the UEFA 2020 analysis only amounts to 1.30 log-likelihood units. We interpret this as the sample containing predictions with *essentially* the same amount of support from the data. Despite this, the range of outcomes predicted is comparatively diverse, and generally contradictory. Furthermore, in the UEFA 2020 uncertainty analysis, the win probabilities for Italy range between less than 0.05 to greater than 0.2. Likewise, the top 5 teams by median win probability in NCAA 2022 have mostly overlapping ranges for estimated win probabilities. In other words, there is a high uncertainty as to which team is the most likely to win. However, the forecast is clearly more certain for the NCAA 2022 sample when compared to the UEFA 2020, despite the smaller number of teams in UEFA 2020. This is due to the NCAA 2022 analysis using substantially more historical matches (on the order of $\approx 40$ times more historical matches). However, NCAA tournaments are an exception, with a large number of teams in each group, and therefore a large number of matches in the lead up to “March Madness”. Furthermore, the amount of data in the UEFA 2020 analysis was intentionally reduced for this work.

From this example, lessons can be learned for the practice of phylogenetics. In particular, care should be taken when analysing a single result from phylogenetic inference, as a given dataset might provide support for a large range of conflicting explanations. For example COVID-19 phylogenies are difficult to estimate for this precise reason (Morel et al. 2021), and placing stock in any single result runs the risk of ignoring other plausible explanations. Therefore, this work is yet another reminder to incorporate uncertainty, particularly of parameter estimates, when performing either phylogenetic or any model based analysis.

## Supplementary Information

Below is the link to the electronic supplementary material.

Supplementary file 1 (pdf 4220 KB)
