# Supplementary material for: Phylourny: efficiently calculating elimination tournament win probabilities via phylogenetic methods
Source: Stat Comput. 2023 May 16;33(4):80. doi: 10.1007/s11222-023-10246-y (PMC10186292; doi:10.1007/s11222-023-10246-y)
Supplement: Supplementary file 1 — (pdf 4220 KB) [file 11222_2023_10246_MOESM1_ESM.pdf]

	Mean	STD	Min	Median	Max	Samples
UEFA	-105.18	2.45	-119.04	-104.99	-97.91	90,000
NCAA	-13842.15	14.00	-13901.66	-13841.72	-13793.86	90,000

Table 1: Summary statistics for the full uncertainty samples. Values shown are Log-Likelihoods of samples taken during the MCMC search for the UEFA 2020 and NCAA 2021 uncertainty analysis.

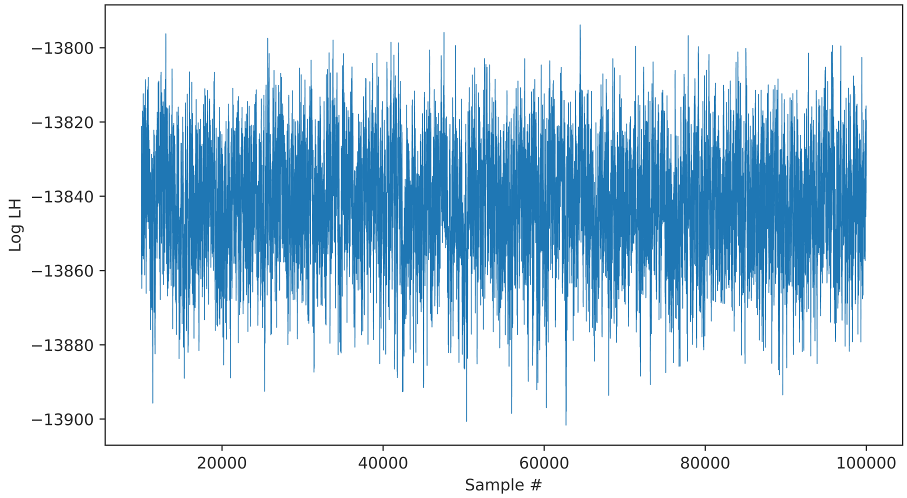

Figure 1: **Trace plot for the MCMC search for NCAA 2022.** First 10% of samples were discarded as burnin for this plot.

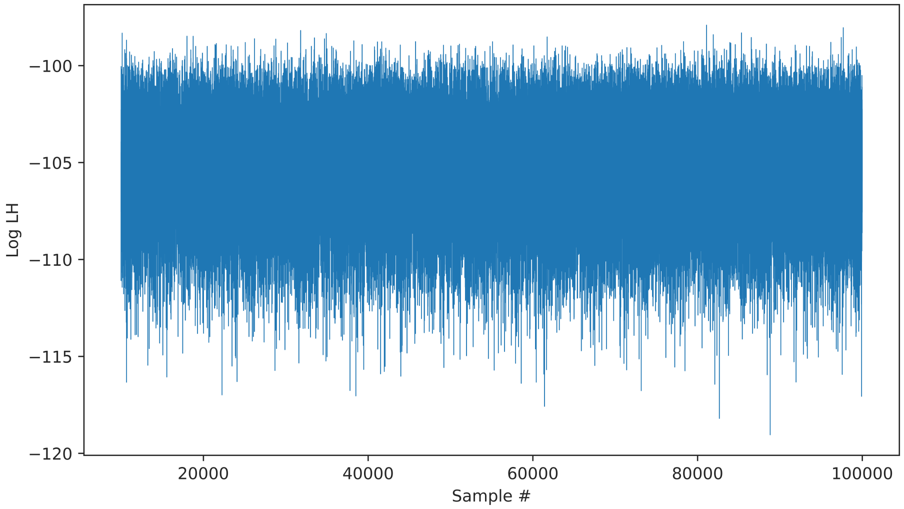

Figure 2: **Trace plot for the MCMC search for UEFA 2020.** First 10% of samples were discarded as burnin for this plot.

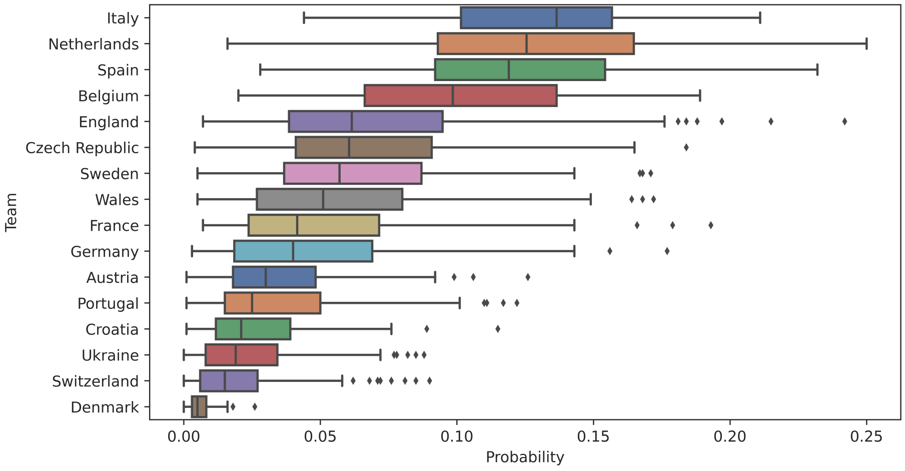

Figure 3: **Win probabilities for the top 99.9% of samples by likelihood for UEFA 2020, computed with simulations**

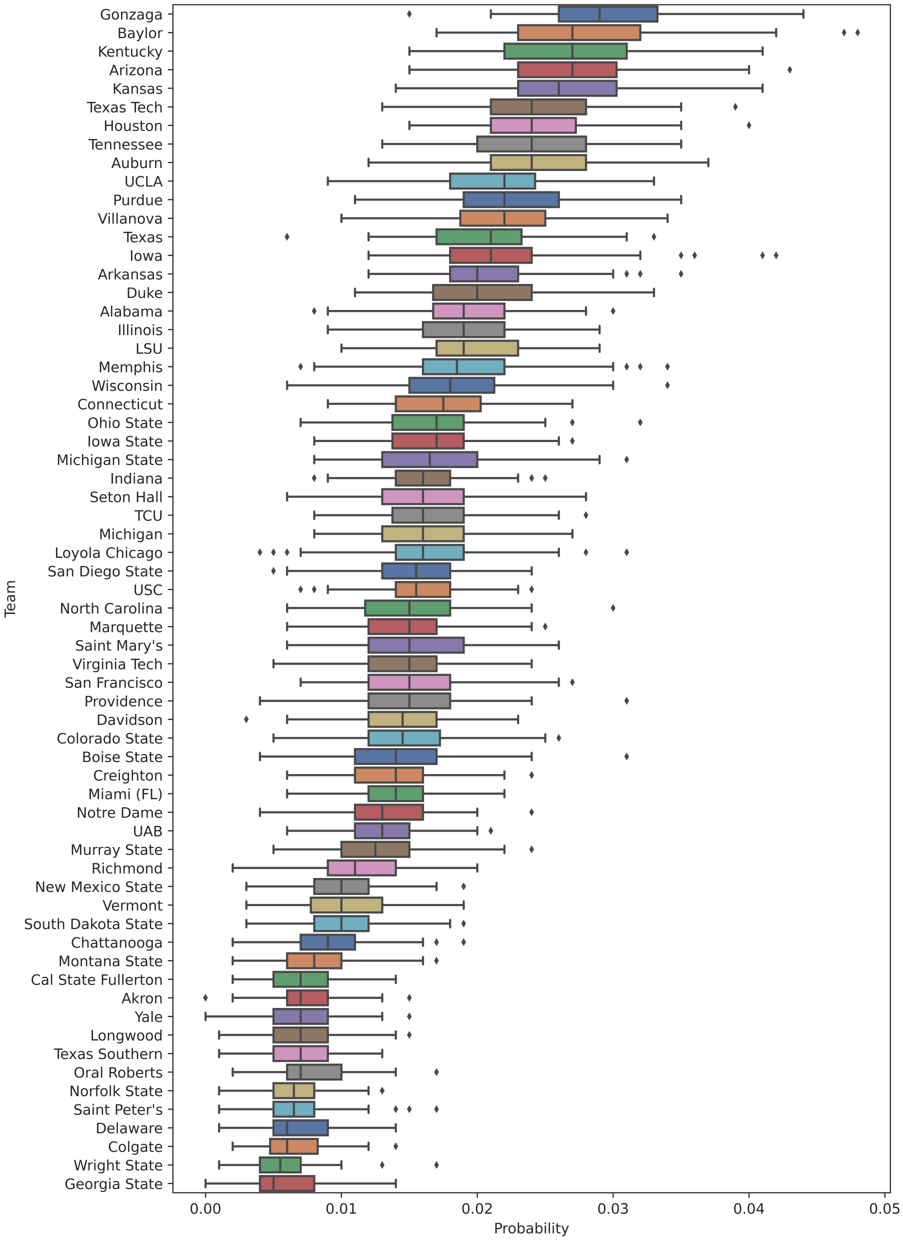

Figure 4: **Win probabilities for the top 99.9% of samples by likelihood for NCAA 2022, computed with simulations.** 1000 simulations per sample was utilized to produce an estimate of the win probability of each team.
